# Formation mechanism and governance strategies of stigma in public health emergencies: Based on event system theory

**DOI:** 10.3389/fpubh.2022.1067693

**Published:** 2023-01-11

**Authors:** Shuzhen Liu, Yuanyuan Liu, Ming Guo, Rui Wang, Qiong Sun, Rong Zhu

**Affiliations:** ^1^Management College, Beijing Union University, Beijing, China; ^2^School of Management and Economics, Beijing Institute of Technology, Beijing, China; ^3^School of Economics and Management, Beijing Jiaotong University, Beijing, China; ^4^Department of Economic Management, Tangshan Normal University, Tangshan, China; ^5^Business School, Beijing Technology and Business University, Beijing, China

**Keywords:** event system theory, public health emergency, stigma, governance strategy, formation mechanism

## Abstract

**Introduction:**

With the new coronavirus (COVID-19) pandemic across the world, it is critical to propose effective strategies for stigma governance in public health emergencies in order to reduce negative effects caused by stigma. However, no known research has focused on the essential role of events in understanding stigma phenomenon from the perspective of external dynamic changes.

**Methods:**

Based on the event system theory, this paper analyzes the evolution mode and characteristics of specific events in the process of stigmatization from strength, space and time aspects, and taking COVID-19 event as an example, 1202 questionnaires and empirical analysis were conducted.

**Results and discussion:**

Our results reveal that event strength directly affects the results of stigmatization, and such impact appears to be more prominent with a novel, disruptive and critical event. In addition, spatial and temporal attributes represent the dynamic development of an event, and they can interact with event strength to regulate the relationship between event strength and outcomes. Finally, stigma governance strategies under public health emergencies from three aspects of event strength, space, and time were put forward.

## Introduction

Ever since the outbreak of COVID-19 at the end of 2019, it has had a great impact on people's lives as well as the economy and society across the world (1). Public health emergencies not only threaten people's physical and mental health and social stability but has a serious subsequent impact of stigmatization (2, 3). Under the COVID-19 pandemic, racism is spreading widely with substantial discrimination and statements against Wuhan, China, and Asians all over the world (4, 5). Historically, in addition to this event, many other pandemics have led to the stigmatization of some populations or regions. The association is always recognized between large-scale viral infectious diseases and their first outbreak region, location, or areas, such as the Surat plague, Spanish flu, Middle-East respiratory syndrome, and Zika virus. Stigma is embedded in social relationships and is constructed in and through daily interactions in social environments (6, 7). Stigma is a social structure that transcends individual attributes and is defined as an undesirable trait, characteristic, or attribute that separates an individual or group from others. It is usually manifested through labeling, stereotyping, avoidance, alienation, and social exclusion, and in extreme cases can also lead to violent behavior (8). In the past, stigmatization and subsequent social avoidance might help reduce the spread of infectious diseases in terms of isolating patients and specific areas. However, in today's context of economic globalization, stigmatization, and the fear of it will significantly accelerate the spread of emerging infectious diseases, which makes prevention and treatment difficult for public health departments. For example, stigmatized individuals or populations are prone to extensive concealment and less medical-seeking behaviors which could badly delay and affect the detection and isolation of close contacts, and could influence the effective allocation of resources regarding infectious disease prevention and control (9, 10). To decrease the potential economic consequences of stigmatization, authorities can suppress information about emerging infectious diseases in their jurisdictions. Although the phenomenon of stigma is evident during public health emergencies, studies have shown that the impact it brings is often more sustained. Siu (11) tracked SARS victims in Hong Kong and found that SARS-related stigma did not decrease over time, and victims continued to suffer from the derogatory effects in contemporary Hong Kong society (11).

It is thus clear that the consequences of stigma go far beyond the direct damage to people's health or living environment, which also prompts scholars to search for the root causes and measures to overcome stigma from multiple perspectives such as sociology and psychology (12–16). However, most current research has focused on the internal stability characteristics of entities, such as the fear of infectious diseases will aggravate the exclusion and avoidance of individuals from others, and the lag in countermeasures will trigger public blame and dissatisfaction with organizational structures. Correspondingly, measures such as increasing the dissemination of disease-related knowledge and improving media risk communication ability can effectively reduce people's fear of unknown diseases and promote the public to think more rationally. But the manner in which stigma governance is based on stable characteristics does not take external variation factors into account, and the effectiveness of stigma governance strategies is usually interrupted by a variety of different events. For instance, large-scale infectious diseases have had a heavy impact on the economy in terms of forced layoffs, which cut off the source of the stable life guarantee for individuals. At this time, the original rational understanding and sympathy for the disease are more likely to transform into irrational emotions of anger and accusation (17). In other words, the generation of stigma is not certain, but not everyone who trends on advantages and avoids disadvantages will discriminate against the victims and regions in public health emergencies, given that the experienced external events contribute to changes in people's thoughts, emotions, and actions. Event system theory (EST) breaks through the limitation of the research perspective of internal stability characteristics of entities and reveals the changes of individuals or organizations after encountering events from the process perspective of dynamic events (18). At the same time, the research on the causes of stigma during public health emergencies is a mostly qualitative theoretical discussion or simple investigation, lacking empirical robustness.

Therefore, this paper explores the formation mechanism of stigma based on event system theory and analyzes the influence of specific events and their consequent events in public health emergencies, to describe the intrinsic multi-level characteristics and time dynamics of related phenomena under public health emergencies. The paper also proposes stigma governance strategies during public health emergencies from the perspective of event management and control.

## Review of relevant studies

### Stigma

Stigma originated in ancient Greece and referred to the permanent imprint on the bodies of criminals, traitors, or slaves to indicate they are undesirable (19, 20). In modern times, stigma is often defined as an undesirable trait, characteristic, or attribute that isolates individuals or groups from others (21). Despite stigma being commonly conceptualized from a psychosocial perspective, such a concept of being viewed as discriminatory or stereotypical has been the core of medical social science research since the 1960s, especially in recent years. Stigma is particularly used to describe the process of negative discrimination against people with certain physical, behavioral, or social attributes in cases of the social impact of disability, mental illness, racial and gender differences in health care, and cultural construction of biomedicine (22, 23).

Fears of specific populations and disease itself can lead to social stigmatization, such as plague patients were stigmatized in the Middle Ages. Similarly, acquired immunodeficiency syndrome (AIDS) has been one of the most scared and imprinted diseased in recent decades. Stigmatization can result in additional adverse consequences of the disease in many ways. First, stigmatization can greatly increase the suffering of the patients. When a person is aware of a negative stereotype and applies it to him/herself, an intrinsic sense of stigma arises which often leads to emotional distress, hopelessness, depression, reduction in self-esteem and self-efficacy, reduced social networks, aversion to access mental health services, etc., (24–27). Previous research identified that individuals and families who suffered from SARS faced various complex problems including physical illness, psychological stress, and financial troubles in addition to social stigma (28). Such issues were experienced and documented by the Chinese community in Chinatown in New York City, who were labeled dangerous, sick, and inferior. Second, patients or people at risk may avoid seeking healthcare services, which makes it more difficult for public health authorities to control the disease. Third, it is possible for professionals and volunteers in the field to be stigmatized, resulting in higher rates of stress and burnout. Finally, stigmatization can contribute to serious economic losses since people may deliberately keep away from specific populations or geographic areas associated with the disease. A study related to hospital visits in Taiwan showed that the fear of SARS and potential hospital infection was responsible for a 23.9% reduction in outpatient care, a 35.2% reduction in inpatient care, and a 16.7% reduction in dental care (29).

Considering the substantial adverse effects caused by stigmatization, scholars have analyzed the causes of stigma from sociological and psychological perspectives. From the sociological point of view, the nature of stigma generation during public health emergencies can be understood from two aspects. On one hand, stigmatization, a means of self-protection for the public, comes from the unknown. When faced with an unknown novel disease, the public may find it beneficial to protect themselves by othering a minority and establishing a “us” vs. “them” narrative (30). Tenkorang (12) interviewed more than 800 individuals from 40 communities in Ghana and found that more than 30% were reluctant to accept Ebola virus survivors to return to their families and communities because they perceived that the survivors might put them at risk of Ebola infection (12). On the other hand, the stigmatization of key persons or institutions is one way for the public to express dissatisfaction. In the face of public health emergencies, there could be a rapid spread of extreme emotions within the society such as anxiety, nervousness, and disappointment due to unmet informed consent, key information being blocked, ambiguous information and explanation, and unsatisfied prevention and management. There appears a loss of foundation in rational judgment resulting in the derogation of the reputation and image of the nodal organizations in charge of managing the health crisis (13).

From a psychological point of view, the generation of stigma is associated with demographic factors such as education level, gender, age, and employment status. Des Jarlais et al. (14) found that the level of education was strongly linked with stigmatization behaviors; while individual negative emotions could cause stigmatizing reactions, education acts as a mediator (14). It is also worth noting that when asked if gay men or Chinese people should be compulsorily examined for AIDS or SARS, 77.9% of respondents with under high school degree agreed, while 17.4% of respondents with graduate degree respondents agreed likewise (15). Previous studies on AIDS, SARS, and tuberculosis have also indicated a potential association between stigma and the attribution of infectious diseases. The allocation of individual causes of disease such as controllability and responsibility affects people's emotional and behavioral responses to disease carriers. For instance, when the public believes that the infection can be controlled by individuals, patients may be required to be responsible for their infections, and thus will be blamed and rejected by society. Lee et al. (16) analyzed the public attitudes toward SARS and concluded that stigma and its management strategies varied with gender, age, education level, employment, proximity to the onset place, and other SARS risk factors (16). Respondents who were middle-aged, high-income, employed, and worried about SARS infection had a significantly increased chance of having a stigmatizing attitude; within this demography, housewives reported the most avoidant behaviors for SARA patients. Tenkorang (12) also proved that individual- and community-level factors were significant determinants of stigmatization (12). Respondents who believed in Ebola rumors were more likely to endorse Ebola-related stigma. Similarly, those who were worried about the possible outbreak, had moderate awareness of Ebola risk, had primary and secondary school education, were not confident of the quality of medical care, and were prone to agree with Ebola-related stigma.

Studies on the mechanism of stigma generation could shed light on the development of guidelines for anti-stigma programs and public health interventions. Currently, most public education initiatives focus on the dissemination of knowledge to enhance public awareness and understanding of the disease while reducing public bias (31). Besides, enhancing media's knowledge and ability to better assess risk and articulate it can reduce people's fear of accidents and unnecessary worries at critical times (32). However, the above stigma management strategies are all based on countering it while ignoring the generation of stigma which is related to specific events which also play an essential role in promoting the process of stigmatization.

### Event system theory (EST)

At present, the majority of social science research is based on variance-oriented theory, focusing on the relatively prominent, persistent, and stable representative characteristics of individuals, teams, departments, organizations, environments, and other entities, and considering these as key reasons leading to changes in organizations, individual attitudes, and behaviors. Characteristics reflect the overall stability of a specific variable of an individual, a group, or an organization, such as individual personality, satisfaction, and work autonomy, and the atmosphere of the organization, reputation, and cultural values. There is no doubt that the characteristics of entities are crucial, but feature-oriented studies focus on the number of features and covariates between multiple features while usually ignoring the impact of dynamic events on entity change and development (33, 34).

Life consists of various and continuous events which are often taken as the center of their character, environment, and development when people describe their lives. Daily ordinary life is always interrupted by unique, limited, highly emotional, and influential events that occur over time, which play an important role in shaping thoughts, feelings, and actions. Morgeson et al. (18) proposed the event system theory (EST) to integrate a variation-oriented research paradigm focusing on the internal stability characteristics of entities with a process-oriented research paradigm focusing on the dynamic events experienced by entities (18). EST holds that novel, disruptive, or critical events have sufficient influence to produce changes in subsequent events over time, ultimately driving structural changes in organizations and the formation of new organizational norms. In this way, events can form a chain of events and affect systems across time (35). For example, the occurrence of public security emergencies may prompt the reform of the safety emergency systems (36). Similarly, the positive emotions of entrepreneurs in the financing processes are related to the financial support received (37), and high-intensity group turnover events can have an impact on work objectives, motivation, and organizational stability of the next retainers (38, 39). Another example is different types of large events (e.g., Olympic Games, Super Bowl, political conferences) and natural disasters (e.g., floods and hurricanes) which have an impact on the philanthropy of US businesses (40).

Events differ from entities in many ways, among which the biggest difference being that events are discrete and bounded in space and time. Events have a clear, identifiable start and end time, while entities are continuous and have a continuously stable mode of existence (41). EST explains events from three perspectives: (a) event strength (novelty, disruption, and criticality); (b) event space (how to propagate the origin of events and their resulting effects); (c) event time (occurring time, time for events to remain influential, and evolution of event strength). Even if some events occur only for a brief period of time, they may permanently change existing functions of the work environment or generate new functions. For example, EST has provided a reference to work-life event theory by explaining how couples cope with work-life shock events (33). In terms of EST, Liu et al. (42) emphasized that situational events faced by enterprises and CEOs are able to change the emotion and cognition of CEOs, the mediation of the senior management team, and organizational processes, and thus made the influence CEOs had on enterprise performance stronger or weaker (42). Thus, EST-based analysis is able to demonstrate how specific events lead to stigma generation during public health emergencies from the three aspects of event strength, space, and time.

## Stigma formation process during public health emergencies based on EST

### Event strength

We encounter a wide variety of events every day, but not all events require attention. In the specific context of EST, events refer to things that interrupt the life and routine of an organization and rapidly control information processing (43, 44). Some researchers also refer to events as “problems”, “fires”, “shocks”, “surprises”, and “events” (45–47). EST identifies what kind of events need attention and will produce changes and examines them against the three dimensions of novelty, disruption, and criticality.

Event novelty, a symbol of a novel or unexpected phenomenon, reflects the extent of difference between current and past behaviors, characteristics, and events. Novel events are always unexpected, unconventional, rare, and surprising, and break people's expectations. When an event is novel, the entity usually does not have enough rules or procedures to effectively respond to the event, and novel events require the entity to change or create new behaviors or ways of thinking to respond to the event, thus attracting more and more attention from the entity (48). Experience from the COVID-19 outbreak showed that it didn't attract much attention in the early stages because of the frequent regional infectious diseases. However, when people became aware of its difference from other well-known diseases in terms of droplet transmission and long incubation period, they panicked and extensively discussed the virus and its characteristics (49).

Event disruption refers to environmental discontinuity caused by the change in the external environment, which is related to the amount or degree of change in daily activities and reflects the destructive and dramatic nature of the event. The occurrence of disruptive events halts the originally planned activity, further prevents or changes ongoing routines, and requires the entity to adjust and adapt, as well as engage in more meticulous and laborious information processing, and change existing behaviors, functions, or create new ways of behavior (50). For example, the public health emergency caused by COVID-19 was disruptive and challenged people's traditional thoughts and response patterns and forced individuals or organizations to make changes, such as home isolation policy, working mode adjustment, and discontinuation of various commercial and business activities that affected people's live and corporate economic status.

Event criticality reflects the importance, necessity, or priority of an event, and often triggers additional analysis and changes in entities. Entities generally do not invest valuable resources and effort to explain and deal with ordinary or trivial things; however, the more critical the event is, the more likely it is to be seen as a prominent event and the more unusual attention and action are required. Critical events tend to attract widespread attention and influence the allocation of resources. When events are critical, new behaviors, properties, and processes are more likely to emerge. Conversely, when events are not critical, entities may not notice them or respond to them. For instance, the announcement of a series of prevention and control measures at the national level, and the support and allocation of national medical resources to Hubei Province reveals that the COVID-19 outbreak is a critical event.

### Event space

Event space refers to the specific location of the origin of events and the way in which the effects of events propagate and spread in the organization, which reflects the multi-level nature of event effects and indicates how the effects of events move in the organizational space. Events tend to occur at specific locations, places, and hierarchies with downward, upward, or internal impacts. Considering the mobility of events, the moderating effect of event spatial properties on event strength and outcomes are described below with factors of event spatial direction, event origin, event spatial dispersion, and event spatial proximity.

#### Event spatial direction

**Figure 1:**
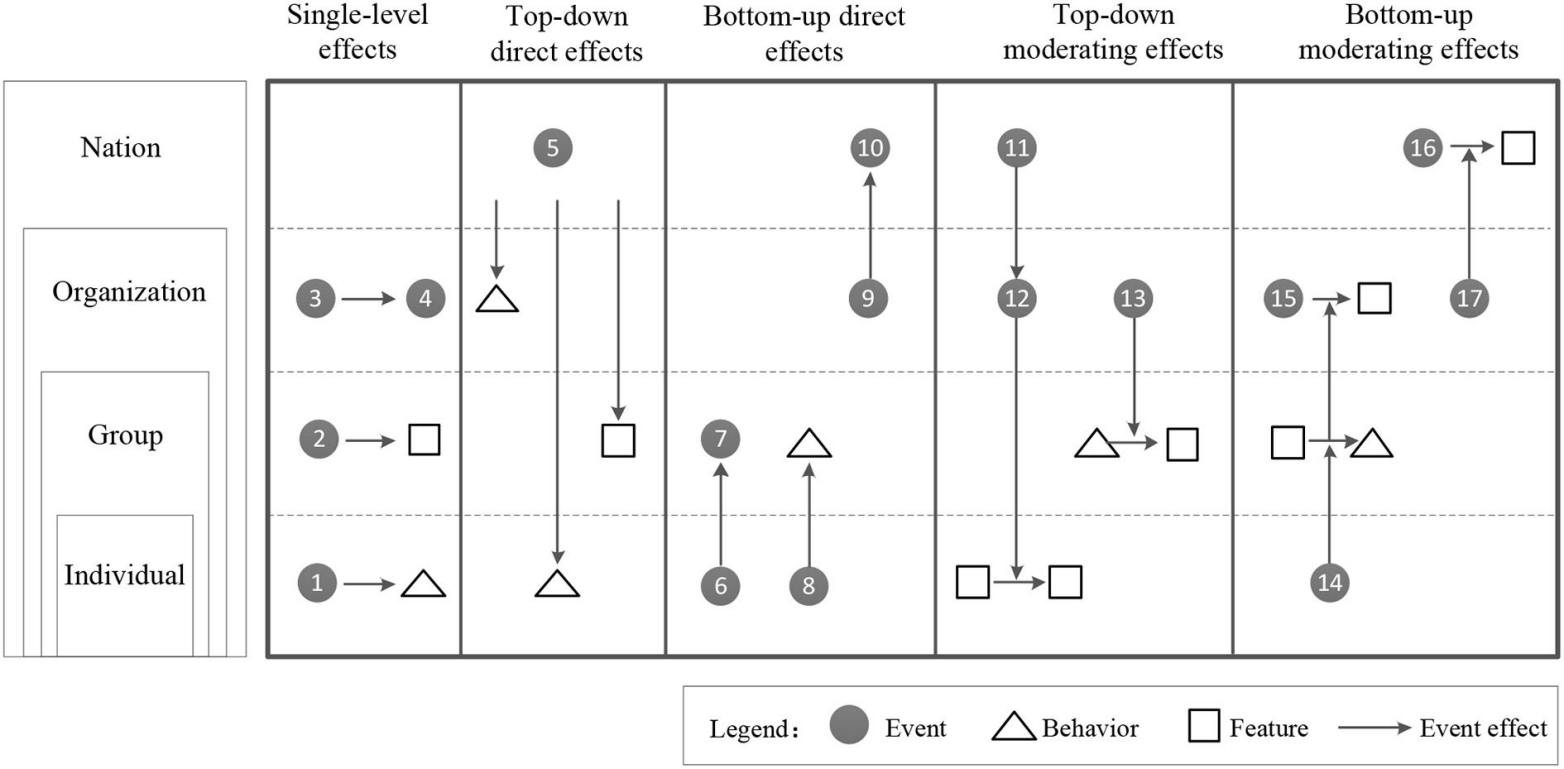
Typical effects of events on entities in the spatial direction. Typical cases are only illustrative. The numbers of each event in the figure correspond to examples of case numbers used in this article.

Figure 1 shows five typical forms of effects, indicating the propagation of events and their resulting effects within or across different tiers. According to the influenced coverage of public health emergencies, this study divided the dissemination hierarchy into four levels: individual, group, organization or department, and national or international.

**Single hierarchy effect**. Events can have a single hierarch effect on behaviors, characteristics, or subsequent events, that is, both occurrence and impact effects of events are limited to the same level. The effect of events has attracted scholars' attention to events of a given grade and their impact on the results of the same grade. At the individual level, the *Business Insider* reported a fight on the New York subway after the H1N1 outbreak because a woman didn't cover her mouth when she sneezed (event 1). At the group level, during the avian influenza outbreak in Surat, India, in 2006, 500,000 people fled from the city fearing a similar situation as the 1994 bubonic plague in the same city (event 2), which directly led to the exclusion of many of these people from adjacent communities and cities. At the organizational level, early reporting by communities may be hampered due to concerns about the economic impact of stigmatization. For instance, in organizations at the highest risk of infection during pandemics, such as in Thailand, there have been significant delays in avian influenza reporting (event 3). To this end, a conference on social justice and influenza was held in Bellagio, Italy, in 2006, which produced a six-point “statement of principles” on issues related to fear of discrimination, retaliation, and uncompensated loss of livelihood, pointing out that “we need to make special efforts to promote reports by disadvantaged groups and protect them from the negative effects that could worsen their situation”.

**Direct bottom-up or top-down effect**. The event itself can have a direct top-down effect on behaviors, characteristics, and subsequent events at lower levels, or a direct bottom-up effect at higher levels. These direct bottom-up effects, as the main way to produce collective phenomena, represent how events at lower levels result in behavioral changes, new features, or the occurrence of subsequent events at higher organization levels. And with individual and collective interactions, such effects further create larger collective structures. For instance, following the avian influenza outbreak in Surat, India, in 2006 (event 5), social reforms were carried out in terms of outstanding improvements in hospital health facilities and primary healthcare delivery, and improvement in the relationship between Hindu and Muslim communities in the city, which in turn increased public confidence in health authorities and their ability to respond. These measures helped reduce public stigma and anxiety in emergencies. During the COVID-19 pandemic, a few infected overseas students concealed their condition (event 6) which led to cross-infection (event 7) on the flight on their way back to their country, which not only aggravated the work burden of customs inspectors but also triggered intense anger against overseas returnees. Furthermore, the then US President Donald Trump used highly stigmatized and discriminatory words such as “Chinese virus” and “foreign virus” on many public occasions (event 8), which catalyzed xenophobia in parts of the US population, further contributing to the recent surge in hate-crimes against Asians. This lends evidence to the notion that opinion leaders usually have certain supporters, and their comments are often contagious to the whole group. During the COVID-19 outbreak, the disclosure of whereabouts information on social donations was not transparent (event 9), which caused a direct public challenge to the work and management system of the Red Cross Society and a few other charities. It attracted the attention of national and provincial administrations which followed up with investigations and legal action against errant institutions and individuals (event 10).

**Bottom-up or top-down moderating effect**. The event itself can have a moderating bottom-up effect on behaviors, characteristics, and inter-event relationships at lower levels, or a moderating top-down effect at higher levels. Just as many companies started layoffs to reduce losses (event 12) with the virus spreading the world over (event 11), this also affected the psychological security and loyalty of the retained employees. Influenza A (H1N1) first broke out in Mexico, and words such as Mexican flu, swine flu, and Mexican swine flu were often in media reports (event 13). Although it was eventually renamed, the initial widespread geographically-labeled reporting provoked public fear resulting in the marginalization and exclusion of people from Mexico even if they showed only physical symptoms such as common allergies. In the 1976 swine influenza pandemic in the United States, public health officials who urged mass vaccination at the early stages were later criticized and condemned for adverse effects in vaccinated populations (event 14). These accusations made public health officials to be more cautious in later epidemic responses, increasing the likelihood of them ignoring initial small-scale community infections and media reports. It also resulted in them being less vocal in promoting preventive measures during the initial stages of the epidemic as proposed by some departments (event 15), which heightened public doubts and stigma about the ability of the relevant departments to respond. During the H1N1 outbreak in 2009, there were many isolation policies against Mexico in several countries to arrest the spread of infection through the reduction of flights and pork products from Mexico (event 16); there gradually appeared international bias and resistance against Mexico. Given its highly contagious nature, the World Health Organization (WHO) classified H1N1 as a “pandemic” at an emergency meeting (event 17). Despite the original purpose of the WHO to bring the epidemic to the forefront, this decision further amplified Mexico's international reputational damage through the slander and demonization of Mexican migrants. Even healthy Mexicans were forced into isolation and treated as disease carriers.

#### Event origin

The event spatial origin attribute describes how the hierarchical level of event occurrence affects behaviors, characteristics, and events. Events originate from inside or outside the organization with direct or indirect impact on entities. EST suggests that events can occur at any level, but despite having equal event strength, they will have a greater influence on the organization when they occur at higher levels (e.g., health institutions, media reports) than at lower levels (e.g., personal extreme speech). Events at higher organizational hierarchy have a wider range of potential impacts on the overall environment, and shape lower-level behaviors, characteristics, and events. This also means that in public health emergencies, compared with individual-triggered events, organization or group-triggered events are more likely to produce broader stigmatization. As Williams et al. (51) found, media was the driving force of connection and information consumption, but in the face of a sudden public crisis, the irresponsible production and information of media might become a dangerous interfering factor. When reporting H1N1 outbreaks, negative media publicity increased the stigmatization of Mexico without playing a role in raising public awareness of appropriate prevention methods (51).

#### Event spatial dispersion

**Figure 2:**
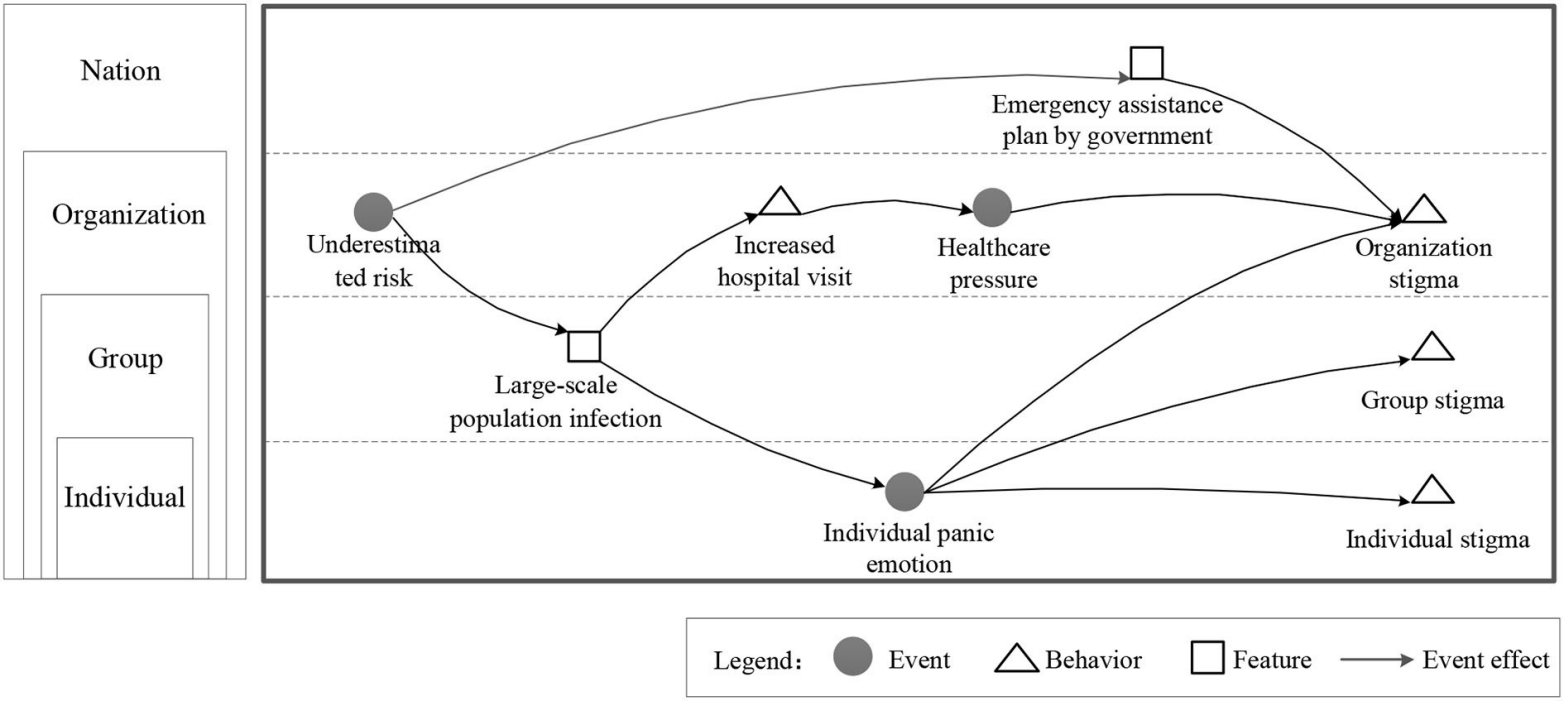
Diffusion effect of events on entities at different levels.

Regardless of the hierarchical structure in which the events occur, the extent to which their effects are dispersed across the organizational hierarchy can vary considerably. Some events appear and remain at the same hierarchical level, but their ability to impact extending over time to other levels is referred to as the event spatial dispersion attribute. EST reports that event strength affects results by interacting with event spatial dispersion. Compared with lower-level affecting events, events that affect higher levels with novelty, disruptiveness, and criticality are more likely to change or create behaviors, characteristics, and events. Like the domino effect, improper handling of one issue can trigger a series of subsequent effects. Figure 2 presents the diffusion effects of events on entities at different levels. At the beginning of the COVID-19 outbreak, health organizations in some countries underestimated the risk. Therefore, the lack of timely prevention and control policies resulted in widespread infection and a heavy burden on healthcare systems due to the surge in hospital visits. As infections and rate of mortality increased, there was panic among the general population which exacerbated the exclusion and stigma toward virus carriers and people from high-incidence areas. Although emergency plans were developed subsequently by governments, the public blame and dissatisfaction with relevant agencies did not reduce because of the unresponsive curb on the spread of disease.

#### Event spatial proximity

Event spatial proximity is used to describe the distance between an entity and where the event occurs. When entities are closer to the event onset location, they may receive information and prompts more directly and effectively, and may also be more strongly affected by events. EST suggests event spatial proximity moderates the relationship between event strength and outcome, thus compared with events farther away from the entity, events closer to the entity with novelty, disruptiveness, and criticality are more at risk to change or create behaviors, characteristics, and events. This also explains the social controversy when there are corporate layoffs. Because of the pandemic, core individuals in the layoffs event will largely lose confidence in enterprises and exacerbate anger at people or organizations that cause or promote the development of disease. On the contrary, employees who are far from layoffs are more understanding and supportive and believe that this is an effective way to respond to economic shocks for their enterprises.

### Event time

An event differs from a work environment with long-term stability characteristics through its limitation in the aspect of time. An event can be transient and have limited effect in time or scope, and it also can be permanent with wider coverage of influence. EST depicts event time attribute in terms of event duration, event timing, and event strength change.

**Event duration**. Despite events being time-limited, their duration may vary. Some events last only a short time while others prolong over time and exert a greater influence. EST believes event duration moderates the relationship between event strength and outcome, thus compared with short-term events, long-term events with novelty, disruptiveness, and criticality are more likely to change or create behaviors, characteristics, and events. Along with the escalated global impact of COVID-19, prevention and control policies in various countries are also continuously extending, which has stirred dissatisfaction and doubts among some sections of the public about the purpose of these government policies. For instance, reports from Austin American-Statesman showed that around 300 demonstrators gathered at the US Capitol building claiming COVID-19 to be a scam and demanding the government reopen the economy.

**Event timing**. Entities such as organizations, teams, and individuals go through different stages of development with different needs. Events that match the development stage of the entity are more likely to trigger a response sufficient to cause changes. EST posits that event timing moderates the relationship between event strength and outcome. Therefore, compared with events that are inconsistent with the development stage of the entity, those events that are consistent with the entity's development and exhibit characteristics of novelty, disruptiveness, and criticality are more likely to change or create behaviors, characteristics, and events. The COVID-19 outbreak coincided with the Chinese Spring Festival which might be the world's largest population migration event. With the travel rush across the country, a strong sense of panic gripped the population, instead of family reunion and festive joy. Social needs were greatly hindered which directed people's anger and accusation toward ground-zero of the epidemic area such that people from that area were isolated from social interactions.

**Event strength change**. The extent to which an entity is affected by an event can be attributed to its overall level (i.e., average event strength over time) and development trend (i.e., event becomes more or less significant over time). In general, it is more possible for an average event strength to affect the outcome if there is a faster growth trajectory, and the converse is also true. EST suggests that event strength changes over time and moderates the relationship between event average strength and outcome, thus the increasing or decreasing event strength over time is more or less likely to change or create behaviors, characteristics, and events. A persuasive example is the initial donation return sheets during COVID-19 which were questioned as falsified, followed by a gag on live media broadcasts and doctors going online seeking help and subsequent related events. All these created negative public opinions on charities which continued to escalate and further destroyed their social credibility.

**Figure 3:**
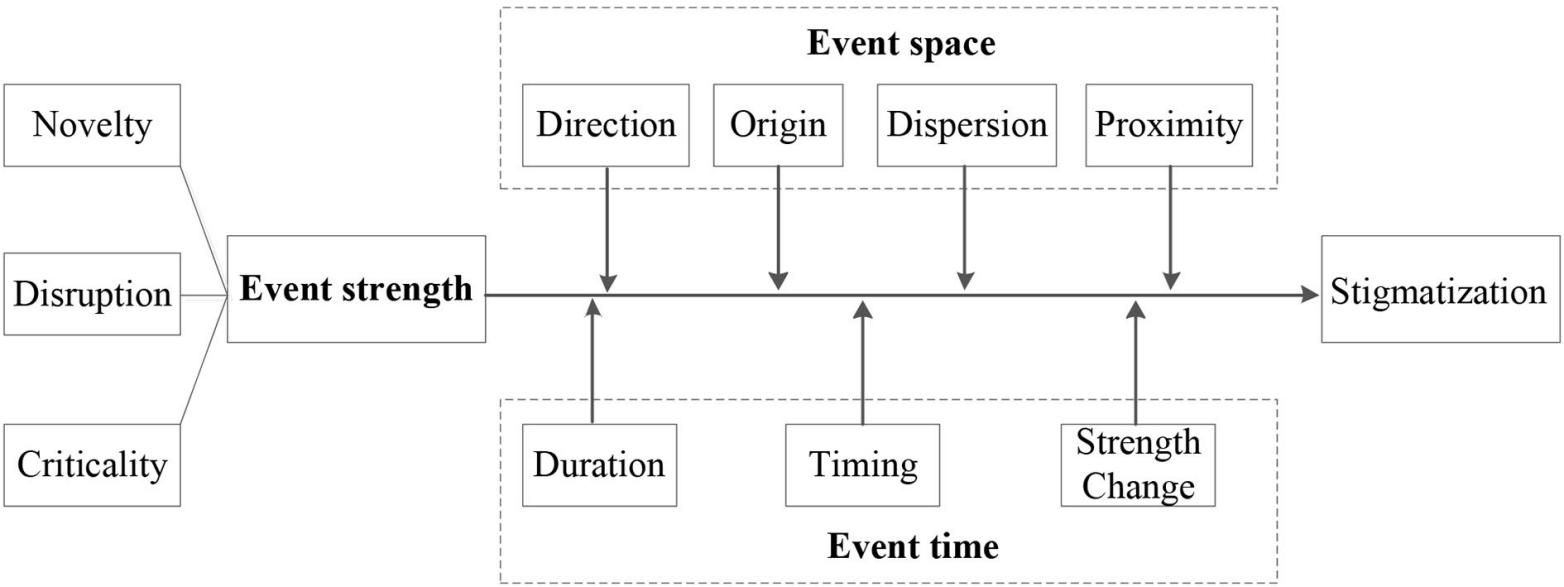
Mechanism of stigma formation based on EST.

Based on the EST, this paper analyzes the transmission and evolution of specific events in public health emergencies to examine the process of stigmatization during public health emergencies, A visual representation of the stigmatization process is shown in Figure 3. The events that trigger the formation of stigma during public health emergencies have three attributes: strength, space, and time. Event strength directly affects the result of stigmatization, while event space and event time moderate the relationship between event strength and stigma. Therefore, the following hypotheses are put forward.

**Hypothesis 1**: Event strength can positively affect the formation of stigma under public health emergencies.
**Hypothesis 2**: Event space moderates the relationship between event strength and stigma under public health emergencies.
**Hypothesis 3**: Event time moderates the relationship between event strength and stigma under public health emergencies.

## Research design and empirical analysis

### Samples

This study took the COVID-19 pandemic as an example to compile a measurement questionnaire and randomly surveyed the population between 8 June and 10 October 2021. We tested the validity of the questionnaire before it was distributed. Since all the mature scales published in top journals were used in this paper, double-blind and standard translation methods were adopted to ensure the consistency of the questionnaire. In addition, well-known domestic management scholars were invited to test and revise all the Chinese scales. Then, the students sent the questionnaire to the employees for a small sample test. The results showed that the reliability and validity of the questionnaire were high and can be further distributed to large samples. A total of 1,256 online questionnaires were collected, excluding those with the same options in a row and with obvious regularities, there were 1,202 valid questionnaires, and the effective recovery rate was 95.70%.

The respondents came from 24 provinces in China, including Beijing, Fujian, Henan, Qinghai, and Hubei. Among the respondents, 58.25% were male and 41.75% were female; in terms of age, 2.66% were 20 years old and below, 41.60% were 21–30 years old, 29.95% were 31–40 years old, 18.80% were 41–50 years old, 51–60 years old accounted for 6.32%, 61 years old and above accounted for 0.67%; in terms of education level, junior high school and above accounted for 3.33%, high school (including secondary technical secondary school and vocational high school) accounted for 5.99%, junior college accounted for 7.65%, undergraduate accounted for 33.78%, and graduate students accounted for 49.25%; in terms of employment type, cadres of state organs accounted for 20.63%, state-owned enterprises accounted for 3.67%, private enterprises accounted for 17.30%, public institutions accounted for 38.77%, unemployed accounted for 11.81%, and others accounted for 7.82%.

### Variable measurements

(1) Events

The characteristics of events in this study include event strength, event space, and event time. The measurement of event strength draws on the event strength scale compiled by Morgeson et al. (18) to measure the strength of the COVID-19 event from three aspects: novelty, disruption, and criticality, and included 11 items (13). For example, “COVID-19 is an unexpected and unconventional event for me,” “COVID-19 is a very important event for my future work and life,” “This COVID-19 changed the way I used to work,” and so on. According to the four characteristics of the event space, namely the direction, origin, dispersion, and proximity, this paper compiled four items for measuring the spatial attributes of the COVID-19 event, such as “In this epidemic, compared with personal information, I pay more attention to the practices and measures of governments, organizations, and institutions,” “COVID-19 has made a great impact on my organization (such as school and work unit),” and so on. Similar to event space, based on the three characteristics of duration, timing, and strength of event time, this paper compiled three items for measuring the time attribute of COVID-19 event, such as “COVID-19 has a greater impact on the Spring Festival,” and “I feel very anxious when the outbreak is just breaking out.” The Cronbach's α values of the event strength, event space, and event time scales in this study were 0.89, 0.91, and 0.88, respectively.

(2) Stigma

Drawing on the AIDS stigma questionnaire compiled by Shrum et al. (52), we selected the items suitable for COVID-19 and changed the relevant situations into expressions suitable for COVID-19. We measured the tendency of the public to stigmatize specific groups during COVID-19. Finally, we got a scale of six items including three dimensions, namely, moral judgment, proximity to fear, and law and society. Some items like “If someone around me gets COVID-19, I will avoid him,” “I think the government should isolate COVID-19 patients or send them to specialized treatment institutions to prevent infection,” and “I am not very willing to contact with people from the epidemic area, even if they are very healthy”. In this study, Cronbach's α value of the scale was 0.92.

(3) Control variables

According to the research on stigma, gender, age, education, and employment among other demographic variables affect stigma (14, 15). Therefore, these four demographic variables were used as control variables in this study.

### Descriptive statistics and confirmatory factor analysis

SPSS 26.0 software was used for the statistical analysis of the variables. The descriptive statistics and correlation coefficients between variables are shown in Table 1. Event strength and stigma (*r* = 0.20, *p* < 0.05), event space and stigma (*r* = 0.38, *p* < 0.05), and event time and stigma (*r* = 0.29, *p* < 0.05) were significantly positively correlated.

**Table 1:** The descriptive statistics and correlation coefficients.

	**M**	**SD**	**1**	**2**	**3**	**4**	**5**	**6**	**7**
1. Gender	1.52	0.50							
2. Age	2.87	1.01	−0.05						
3. Education	4.20	1.04	0.03	−0.18^**^					
4. Employment	4.32	1.05	0.01	0.32^**^	−0.11^*^				
5. Event strength	3.03	0.85	0.03	0.11^**^	−0.07	0.05			
6. Event space	3.16	1.02	0.11^**^	−0.07	0.02	−0.05	0.23^**^		
7. Event time	3.34	0.93	0.12	−0.05	0.04	−0.06	0.31^**^	0.26^**^	
8. Stigma	3.19	0.76	0.01	0.02	0.02	0.07	0.20^**^	0.38^**^	0.29^**^

* *p* < 0.1,

** *p* < 0.05.

Amos 22.0 software was used for confirmatory factor analysis. As shown in Table 2, each fitting index of the four-factor model (χ^2^/df = 1.53, NFI = 0.91, IFI = 0.93, TLI = 0.91, CFI = 0.92, RSEMA = 0.06) was better than that of other models, indicating that the four variables had good discriminant validity.

**Table 2:** Confirmatory factor analysis.

**Model**	**χ^2^/df**	**NFI**	**IFI**	**TLI**	**CFI**	**RMSEA**
One-factor (Strength + Space + Time + Stigma)	7.61	0.70	0.73	0.71	0.72	0.13
Two-factor (Strength + Space + Time, Stigma)	5.33	0.79	0.77	0.76	0.78	0.10
Three-factor 1 (Strength, Space, Time + Stigma)	3.52	0.83	0.82	0.84	0.83	0.09
Three-factor 2 (Strength + Space, Time, Stigma)	3.45	0.82	0.81	0.82	0.82	0.09
Three-factor 3 (Strength + Stigma, Space, Time)	3.65	0.83	0.84	0.82	0.83	0.09
Three-factor 4 (Strength, Space + Time, Stigma)	3.21	0.83	0.82	0.84	0.83	0.09
Four-factor (Strength, Space, Time, Stigma)	1.53	0.91	0.93	0.91	0.92	0.06
Criteria	<3	>0.90	>0.90	>0.90	>0.90	<0.08

### Hypothesis test

The hypotheses were tested by the hierarchical regression method. The results were shown in Table 3. The control variable and independent variable (event strength) were incorporated into the equation to obtain model 2. Event strength had a significant positive impact on stigma (β = 0.38, *p* < 0.05), thus hypothesis 1 was verified. The interaction terms of event strength and event space, event strength, and event time were constructed, respectively and incorporated into the equation to obtain models 3 and 4. It can be seen that event space plays a moderating role in the relationship between event strength and stigma (β = 0.28, *p* < 0.05), and the event time plays a moderating role in the relationship between event strength and stigma (β = 0.18, *p* < 0.05).

**Table 3:** The results of hierarchical regression.

**Variables**	**Stigma**			
	**Model 1**	**Model 2**	**Model 3**	**Model 4**
Gender	0.01	0.01	0.01	0.02
Age	0.01	0.02	0.02	0.01
Education	0.02	0.03	0.02	0.03
Employment	0.02	0.02	0.03	0.02
Event strength		0.38^**^	0.31^**^	0.23^**^
Event space			0.35^**^	
Event time				0.27^**^
Strength^*^Space			0.28^**^	
Strength^*^Time				0.18^**^
R^2^	0.04	0.18	0.43	0.47
ΔR^2^	0.04	0.14	0.39	0.43

* *p* < 0.1,

** *p* < 0.05.

**Figure 4:**
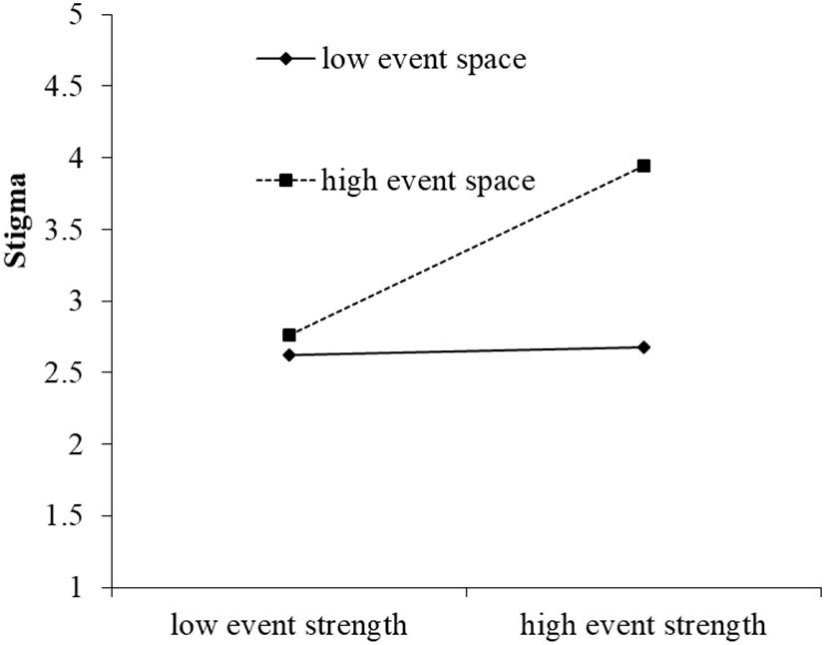
The moderating effect diagram of event space between event strength and stigma.

**Figure 5:**
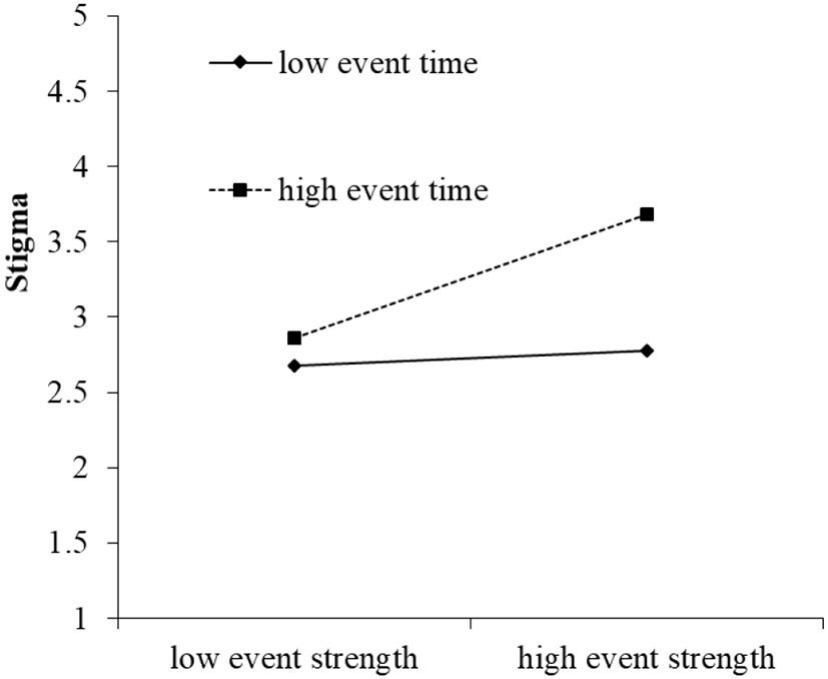
The moderating effect diagram of event time between event strength and stigma.

To further test the moderating effect of event space and event time, the moderating effect diagram of moderating variables in high and low groups (1 standard deviation above and below the mean) was drawn. As shown in Figure 4, in the low event space, the impact of event strength on stigma was not significant (*r* = 0.03, n.s.); in the high event space, the effect of event strength on stigma was significant (*r* = 0.59, *p* < 0.05), so hypothesis 2 was verified. As shown in Figure 5, during low event time, the impact of event strength on stigma was not significant (*r* = 0.05, n.s.); in high event space, the effect of event strength on stigma was significant (*r* = 0.41, *p* < 0.05); so hypothesis 3 was verified.

## EST-based stigma governance strategies under public health emergencies

Stigmatization is a subsequent psychological phenomenon that cannot be ignored in public health emergencies. In this paper, using EST as the analysis framework, we thoroughly discussed the causes of stigmatization during public health emergencies, analyzed the transmission and evolution characteristics of specific outcomes in public health emergencies, and proposed a mechanism diagram of stigma formation. The events that promote the formation of stigma under public health emergencies have three attributes which are strength, space, and time. Event strength directly affects the outcome of stigmatization, while event space and time represent the dynamics of event development moderating the relationship between event strength and outcome through interactions. An empirical test was carried out with 1,202 samples from 24 provinces in China. Finally, combined with EST and previous related research, we concluded EST-based stigma governance strategies.

### Stigma governance based on event strength

From the aspect of event strength, it is composed of three attributes—novelty, disruptiveness, and criticality. Therefore, evaluations of event effects were needed from these three dimensions in the process of stigma governance. In particular, high-strength events with all these three attributes during public health emergencies should be focused on during stigma management as they are more likely to trigger a wider stigmatization effect. First, long-term coordination and supervision among public health departments, government departments, non-profit organizations, and media are needed for a quick coordinated response during public health emergencies in terms of “found at source and nipped in the bud”. Especially for high-strength events with novelty, disruptiveness, and criticality, active risk estimation is required in advance to comprehensively improve the safety prevention ability through the preventive public health and epidemic prevention system. Second, well-prepared emergency plans should be developed for high-strength events under public health emergencies, such as the expected economic collapse, public security confusion, food and fuel shortage, violence, and uncivilized conditions. Plans should include full and reasonable allocation of emergency material reserve, effective policies to benefit people, and timely response to public social concerns to reduce public panic and anxiety during public health emergencies with professional emergency response-ability. Last, public health systems must be improved by increasing available infrastructure and expanding the availability of medical staff, to effectively enhance public confidence in responding to public health events and reducing stigmatization.

### Stigma governance based on event space

From the aspect of event space, in addition to paying special attention to high-strength events, stigma handlers should also try to avoid the spread of events to other levels, especially to high-level events like the organizational level, since such events are more destructive. First, efforts should be made to reduce the occurrence of high-level negative events by cutting down on negative publicity events in the media. The focus should be on promoting good public opinion and fully utilizing the popularity of the media to effectively disseminate accurate public health information. This will help people understand the transmission of the disease and adopt self-protective measures. Informed publicity will also provide the necessary information on risk evaluation and reduce biased behaviors (53). Simultaneously, attention should be paid to strengthening the monitoring of enterprises at the core of the event, publicly handling the process, and accepting public supervision to avoid the occurrence of events affecting the credibility of relevant institutions (54). Second, the relevant authorities should respond and accept responsibility immediately after high-level negative events to avoid further horizontal and vertical expansion of the events. Finally, with the consideration of event spatial proximity, customized governance strategies should be adopted for groups at different distances from the events. For groups less affected by the events, extensive publicity and education are suggested to increase their awareness about prevention and scientific understanding of the disease. Efforts should be made to include public participation in the process of stigmatization governance and obtain their actual support and understanding. While for groups more affected by the events, effective support measures are needed such as compensation to the unemployed and tax reduction to small- and medium-sized enterprises with economic losses. Overall, the aim should be to minimize the impact of public health emergencies and avoid the spread of negative emotions and the formation of stigma.

### Stigma governance based on event time

From the aspect of event time, longitudinal consideration of the impact of each event during public health emergencies should be made from the perspective of event chains and sets. Stigma events in a chain or set should be taken more seriously by stigma handlers, along with event duration and strength change (55). First, attempts must be made to reduce the event duration. After the organizational stigma event, the relevant institutions should actively assume the corresponding responsibilities to avoid oppositional behaviors that are more likely to intensify public protest (56). Importance must be attached to and given priority to the events in the event chain or set, the veracity of new information must be investigated, public opinion must be guided promptly, and the dissemination time of organizational stigmatization events must be shortened. Moreover, the enhancement of event strength should be avoided. During the critical period before the formation of geographical stigma, group stigma, or organizational stigma, active and effective measures ought to be implemented to stop the continuous fermentation of negative events of malicious attacks on regions, groups, and organizations, which not only inhibit the formation of stigma, but prevent the stigmatization of epidemic areas, affected individuals, and relevant organizations even after the formation of stigma representations. Finally, focus is required on the event timing. The nature of stigma in public health emergencies and its formation process show that the stigmatization effect is an inevitable response of social groups to secure their own interests and needs to insulate themselves from the risks, instead of simple irrational behavior. Stigma handlers should propose a more effective and reasonable governance plan based on fully understanding the background of each event and public appeal.

## Conclusion

Public health emergencies not only threaten people's physical and mental health, and social stability, but have a serious subsequent influence of stigmatization. It is critical to propose effective strategies for stigma governance as part of public health emergencies to reduce the negative effects caused by stigma. However, no known research has focused on the essential role of events in understanding the phenomenon of stigma from the perspective of external dynamic changes. In this paper, using EST as an analytic framework, we thoroughly discussed the causes of stigmatization in the consequences of public health emergencies, analyzed transmission and evolution characteristics of specific outcomes in public health emergencies, and proposed the pathways of stigma formation, including a visual representation of the pathways. Our results reveal that event strength directly affects the results of stigmatization, and such impact appears to be more prominent with a novel, disruptive, and critical event. In addition, spatial and temporal attributes represent the dynamic development of an event, and they can interact with event strength to regulate the relationship between event strength and outcomes. Finally, stigma governance strategies under public health emergencies from three aspects of event strength, space, and time were put forward.

## Data availability statement

The raw data supporting the conclusions of this article will be made available by the authors, without undue reservation.

## Ethics statement

The study involving human participants was reviewed and approved by the Ethical Review Board of the Beijing Union University (Grant: JG20220515). The participants provided their written informed consent to participate in this.

## Author contributions

SL and QS contributed to the idea and wrote the full manuscript. YL and MG collected the data and run the data. RW and RZ revised the full manuscript and proposed improvements. All authors reviewed the manuscript. All authors contributed to the article and approved the submitted version.
